# Efficacy of T Regulatory Cells, Th17 Cells and the Associated Markers in Monitoring Tuberculosis Treatment Response

**DOI:** 10.3389/fimmu.2018.00157

**Published:** 2018-02-05

**Authors:** Sonali Agrawal, Om Parkash, Alangudi Natarajan Palaniappan, Ashok Kumar Bhatia, Santosh Kumar, Devendra Singh Chauhan, M. Madhan Kumar

**Affiliations:** ^1^Department of Immunology, National JALMA Institute for Leprosy and Other Mycobacterial Diseases, Agra, India; ^2^Department of Clinic, National Institute for Research in Tuberculosis, Chennai, India; ^3^Department of Biotechnology, GLA University, Mathura, India; ^4^Department of Tuberculosis and Chest Diseases, Sarojini Naidu Medical College, Agra, India; ^5^Department of Microbiology and Molecular Biology, National JALMA Institute for Leprosy and Other Mycobacterial Diseases, Agra, India

**Keywords:** tuberculosis, Treg, Th17, treatment, monitoring

## Abstract

Treatment monitoring is an essential aspect for tuberculosis (TB) disease management. Sputum smear microscopy is the only available tool for monitoring, but it suffers from demerits. Therefore, we sought to evaluate markers and cellular subsets of T regulatory (Treg) cells and T helper (Th) 17 cells in pulmonary TB patients (PTB) for TB treatment monitoring. Peripheral blood mononuclear cells (PBMCs) were stimulated *in vitro* (with purified protein derivative (PPD)) overnight which was followed by a polychromatic flow cytometry approach to study Treg and Th17 markers and cellular subsets in PTB (*n* = 12) undergoing antituberculous treatment (ATT). The baseline levels of these markers and cellular subsets were evaluated in normal healthy subjects (NHS). We observed a significant decrease in the expression of CD25 (*p*<0.01) marker and percentage of T-cell subsets like CD4^+^CD25^+^ (*p*<0.001) and CD4^+^CD25^+^CD39^+^ (*p*<0.05) at the end of intensive phase (IP) as well as in the continuation phase (CP) of ATT. A decrease in CD25 marker expression and percentage of CD4^+^CD25^+^ T cell subset showed a positive correlation to sputum conversion both in high and low sputum positive PTB. In eight PTB with cavitary lesions, only CD4^+^CD25^+^FoxP3 Treg subset manifested a significant decrease at the end of CP. Thus, results of this study show that CD25 marker and CD4^+^CD25^+^ T cells can serve as better markers for monitoring TB treatment efficacy. The Treg subset CD4^+^CD25^+^FoxP3 may be useful for prediction of favorable response in PTB with extensive lung lesions. However, these findings have to be evaluated in a larger patient cohort.

## Introduction

Tuberculosis (TB) is an indomitable malady for the mankind since time immemorial. The World Health Organization (WHO) has reported 10.4 million new (incident) cases and 1.7 million TB deaths in 2016 (1). In human immunodeficiency virus (HIV) infected individuals, the figures for incidence of new TB cases were 1.04 million and deaths were 0.4 million. Thus, the morbidity and mortality due to TB are of great concern and thus it needs efficient management. Efficient TB management relies on prompt diagnosis and early treatment. During antituberculous treatment (ATT), the sputum microscopy is the yardstick for determining treatment efficacy. It is the only tool available for assessing TB treatment efficacy, but has many demerits (2, 3): (1) Its low sensitivity, i.e., the requirement of 10,000 bacilli/mL of sputum, (2) inability to diagnose smear negative TB, extra pulmonary TB and not of use in those who are unable to produce sputum, and (3) diagnosis is made after a period of 2 months (after completion of intensive phase (IP)) and by that time, the patient would have transmitted the infection, if the person is not responding to ATT. Due to the lack of satisfactory tools for monitoring ATT efficacy, efforts have been undertaken by various research groups to evaluate various immunological markers from different body fluids of host (urine, blood, plasma and serum). Along these lines, this study attempted to investigate the dynamics of T regulatory markers (Treg), Th17 markers and their cellular subsets in pulmonary TB patients (PTB) during ATT for their use in detecting TB treatment efficacy. In TB, Treg are recruited to the site of infection to bring back homeostasis (3). There are many studies that have shown a association between elevated Treg levels and increase in *Mycobacterium tuberculosis* (MTB) load (4–6). In line with this observation, a study has demonstrated the bacterial burden to decrease in lungs upon using chimeric FoxP3 knock-out (KO) mice (7). Similar reduction in bacterial growth has been observed on inhibiting Treg differentiation by using a small molecule (8). Such an immunotherapy led to increase in Th1 responses that protect against MTB infection. This association of bacillary load and Treg prompted us to evaluate the usefulness of Treg markers in treatment monitoring. We also compared the expression of Treg and Th17 markers with bacterial load, sputum conversion and reduction in lung lesions.

## Materials and Methods

### Study Participants

Recruitment of study participants was carried out in National JALMA institute for leprosy and other mycobacterial diseases, (NJIL & OMD) Agra, from March 2015 to April 2017 after obtaining approval from Institutional Human Ethics Committee. All study participants provided written informed consent. The overall recruitment criteria and methodology followed are illustrated in Figure S1 in the Supplementary Material.

#### Pulmonary TB Patients (PTB)

Fifteen individuals ≥18 years of age with newly diagnosed, smear positive PTB (Category I), either naïve for ATT or had received <2 weeks of ATT, were enrolled (Table 1). Active PTB was diagnosed by clinical evaluation, chest X-ray, and positive acid-fast bacilli (AFB) sputum smears. The presence of other immune-suppressive conditions was ruled out by filling out a questionnaire after interrogating the patients about recent organ transplant, cancer treatment, or any type of steroidal treatment. The patients were excluded if they were pregnant or lactating, moribund or had significant liver or renal function abnormalities at baseline, or positive for HIV infection. All the patients had random blood glucose levels in the range of 80–120 mg/dL. HIV testing was done by ELISA, a comb-based assay (Microlisa—HIV, J. Mitra & Co. Pvt. Ltd, New Delhi, India).

**Table 1:** Demographic and clinical details of study subjects.

Characteristic			Number (%)
Participants			15

A. PTB			

Mean age (range, in years)	30 (19–50)		

Sex		Male	10 (67)

		Female	5 (33)

BCG vaccinated			11 (73)

Nutritional deficiency			5 (33)

Random blood sugar (range, in mg/dL)	80–120		

H/o TB in family			9 (60)

Close contact to TB patient			11 (73)

Clinical symptoms		Cough	15 (100)

		Fever	15 (100)

		Night sweats	15 (100)

		Weight loss	15 (100)

		Loss of appetite	15 (100)

		Hemoptysis	6 (40)

Chest X-ray		Opacity	4 (27)

		Opacity with cavitary lesion	8 (53)

B. NHS			17

Mean age (range)	32 (18–58)		

Sex		Male	11 (65)

		Female	6 (35)

BCG vaccinated			17 (100)

Random blood sugar range	80–127		

H/o TB in family			0

QFT positives			7 (41)

*PTB, pulmonary tuberculosis patients; NHS, normal healthy subjects; BCG, Bacillus Calmette–Guérin; H/o TB, history of tuberculosis; QFT, quantiferon; mg/dL, milligram/decilitre*.

The treatment regimen for this group was as per the WHO treatment guidelines (9). Out of 15 recruited PTB, only 12 were followed up for longitudinal studies at different time periods of ATT, i.e., before (zero month, i.e., at recruitment), during (second and fourth month), and at the end of ATT (sixth month). Clinical evaluation was done at every time point of sample collection. Chest X-ray was taken at all time points of follow-up except at fourth month. Peripheral venous blood and sputum were collected from the patients at all time points of follow-up.

#### Normal Healthy Subjects (NHS)

Seventeen age- and gender-matched NHS were recruited for the study (Table 1). These subjects were recruited for only one time point and were not followed up. Clinical TB was ruled out based on the absence of common symptoms of TB (like cough for >2 weeks, fever, fatigue, loss of appetite and weight loss) and by negative sputum smear microscopy. NHS were also tested for sputum smear and culture and were found to be negative. The latent infection in these individuals was ascertained using Quantiferon TB Gold In-Tube assay (QFT-IT; Qiagen, Germany). QFT-IT assay was done as per the manufacturer’s instructions. Irrespective of Quantiferon (QFT) positivity status, all the individuals were recruited for the study (Table 2). Immune-suppressive conditions were ruled out by interrogating the subjects for the above-mentioned conditions (as said for PTB) and filling out a questionnaire. The random blood glucose levels of the NHS were in the normal range (as said above in PTB section). All the NHS were negative for HIV by ELISA (comb-based assay) (Microlisa—HIV, J. Mitra & Co. Pvt. Ltd, New Delhi, India).

**Table 2:** Quantiferon status of NHS.

Categories	Test value^b^ (IU^a^/mL)	Number (%)

Total NHS (*n* = 17)		
High positive	≥10	2 (12)
Positive	≥0.35 to <10	5 (29)
Negative	<0.35	10 (59)

*^a^International units per mL*.

*^b^Cutoff value for quantiferon-TB gold in-tube (QFT-IT) is 0.35 IU/mL. The test values must be ≥0.35 IU/mL for a patient to be considered as positive, if test value <0.35 IU/mL, the persons are deemed negative for MTB infection. In this study, test value ≥10 is considered to be high positive*.

*NHS, normal healthy subjects*.

### Evaluation of Radiological Parameters

The extent of disease was evaluated through chest X-ray scoring done by two independent clinicians who were unaware of the results of immunological assays (Table 3). The parameters which were considered for scoring chest X-rays were as follows: lung involvement (scored as 0—no involvement; 1—unilateral; 2—bilateral involvement), number of lesions (scored as 0—no lesion; 1—single lesion; 2—multiple lesions) and type of lesions (scored as 0—normal; 1—fibrosis; 2—opacity; 3—opacity with single cavity; 4—opacity with multiple cavities). For comparison between clinical and immunological parameters, combination of chest X-ray scores and sputum smear grading were also done. The marker and cellular subset response were studied for all the radiological parameters said above.

**Table 3:** Categorization of PTB based on chest X-ray findings and bacillary load.

Radiological data based on chest X-rays	**Category**	**Subcategory**	**Number of patients (12)**

	Lung involvement	Unilateral	4
		Bilateral	8

	No. of lesion/areas affected	Single	1
		Multiple	11

	Type of lesion	1. Fibrosis	0
		2. Opacity	4
		3. Opacity with single cavity	5
		4. Opacity with multiple cavity	3

	Lung involvement + no. of lesions	1. Unilateral + single lesion	1
		2. Unilateral + multiple lesion	3
		3. Bilateral + multiple lesion	8

	Bacillary load + no. of lesions	1. Low positives + single lesion	1
		2. Low positives + multiple lesions	5
		3. High positives + multiple lesions	6

	Bacillary load + cavity	1. Low positives with no cavity	3
		2. Low positives with cavity	2
		3. High positives with no cavity	1
		4. High positives with cavity	6

*PTB, pulmonary tuberculosis patients*.

### Sputum Preparation for Acid-Fast Bacilli Staining and Culture

Two sputum (spot and early morning) samples were collected from each study participant as per the RNTCP guidelines (10). Modified Petroff’s method (11) was used for sample decontamination and further diagnosis was made based on sputum microscopy by Zeihl–Neelsen staining and MTB culture on Lowenstein–Jensen media.

### Cell Culture and Flow Cytometry

Whole blood was collected from PTB (at all time points of follow-up) and NHS (only once at the time of their recruitment) by venipuncture in heparinized tubes. The blood was layered over ficoll-hypaque (Sigma-Aldrich, St. Louis, MO, USA) and after centrifugation, the peripheral blood mononuclear cells (PBMCs) were collected, washed with Hank’s balanced salt solution (HBSS) (Sigma-Aldrich, St. Louis, MO, USA) and suspended in complete Roswell Park Memorial Institute (RPMI-1640) medium (Sigma-Aldrich, St. Louis, MO, USA) [with 10% Human AB serum (MP Biomedicals, CA, USA)] supplemented with antibiotic antimycotic solution (Sigma-Aldrich, St. Louis, MO, USA). PBMCs were counted and were incubated with or without purified protein derivative (PPD) (10 µg/mL) (Statens Serum Institut, Denmark) overnight in a 5% CO_2_ atmosphere. Brefeldin A (1 µg/mL) (BD Biosciences, CA, USA) was added 16 h before the termination of culture. After culture termination, cells were washed with cold RPMI to remove adherent cells and the fluorochrome-tagged surface labels, such as CD4 PerCP, CD25 FITC, CD39PE, and CD127PE (BD Biosciences, CA, USA) were added and incubated for 30 min at 4°C. This was followed by washing and FoxP3 buffer treatment (BD Biosciences, CA, USA) for fixing and permeabilizing the cells, after which internal labels [i.e., IL-10 PE, TGF-β PE, FoxP3 Alexa fluor@647, IL-17A Alexa fluor@647 (BD Biosciences, CA, USA)] were added and incubated for 30 min at room temperature. Finally, the cells were fixed using 2% paraformaldehyde and acquired by flow cytometer (FACSAria, BD Biosciences, CA, USA). The cells were analyzed using FlowJo v10 software (FlowJo LLC, OR, USA).

### Statistical Analysis

Differences in expression of individual markers [in terms of absolute number, percentage and mean fluorescence intensity (MFI)] and cellular subsets (Treg and Th17 subsets) in blood samples from NHS and PTB were evaluated using Mann–Whitney *U* test. Individual marker expression and cellular subset profiles during the course of TB treatment were analyzed by Kruskal–Wallis test, whereas the paired comparisons between different time points were made by Mann–Whitney *U* test. Association between the bacillary load and expression of markers or cellular subsets were studied using Mann–Whitney *U* test. Marker expression, cellular subset responses, and different radiological parameters were analyzed using Mann–Whitney *U* test. This test was also employed for predicting correlation between marker expression and sputum conversion in the second month. *P*-values <0.05 were considered significant. The data were analyzed using Graphpad Prism software (Graphpad Software Inc., version 5, CA, USA).

## Results

### Patient Follow-up and Outcome

**Figure 1:**
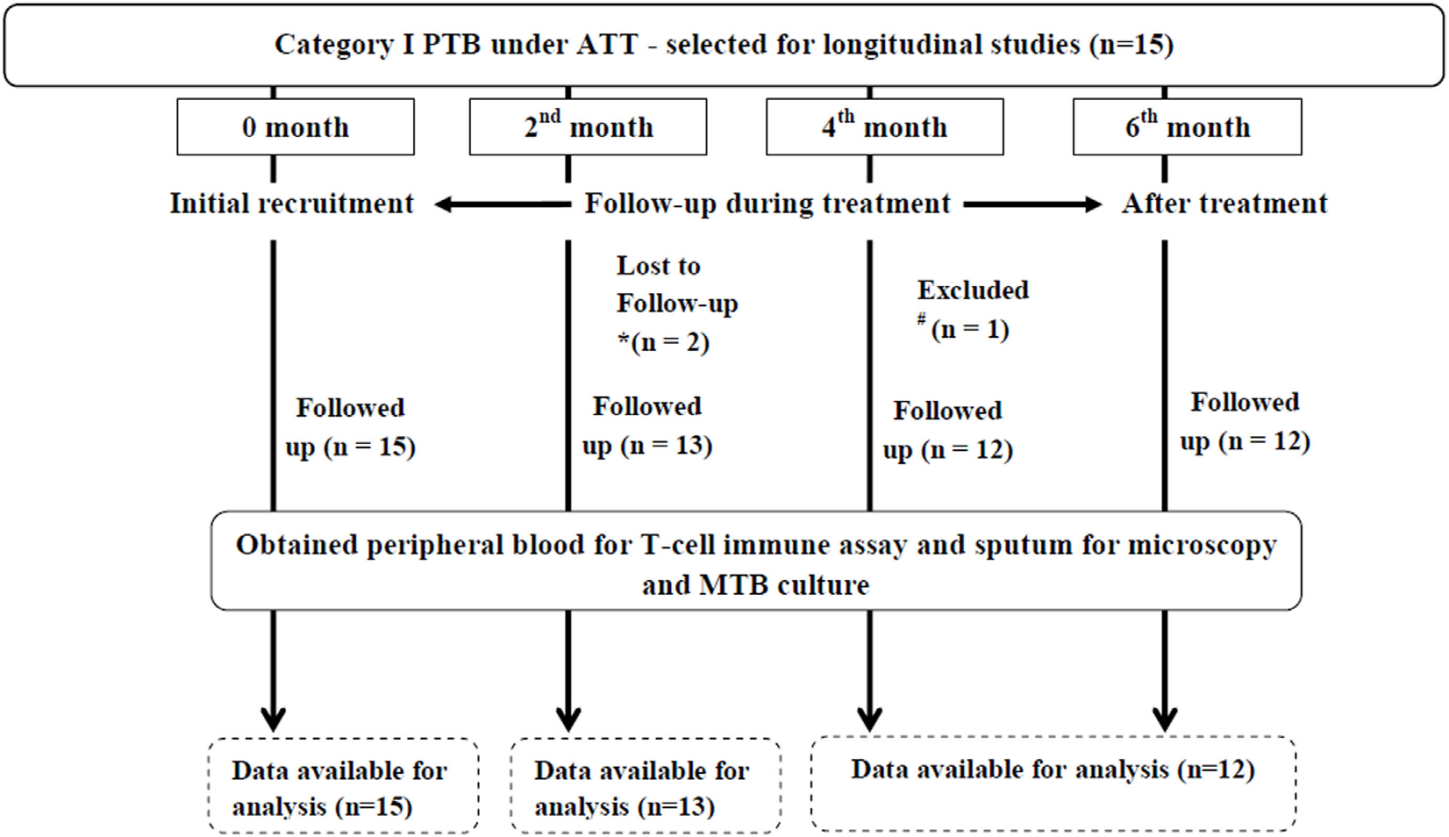
Flowchart depicting longitudinal studies in PTB. Initially, 15 PTB were recruited for longitudinal studies of which two were lost to follow-up due to *non-compliance of patients (at second month of ATT) and one was ^#^excluded due to pregnancy (at fourth month of ATT). Therefore, the longitudinal studies were carried out in 12 PTB. PTB, pulmonary TB patients; ATT, antituberculous treatment; MTB, *Mycobacterium tuberculosis*.

Of 15 enrolled PTB, only 12 were followed up at different time points of ATT (0, 2, 4, and 6 months) for longitudinal studies (two were lost to follow-up and one was excluded due to patient’s pregnancy at fourth month of ATT) (Figure 1). All the follow-up PTB who were initially positive for sputum smear microscopy and culture turned out negative at second month except two (Table 4). Except one PTB who turned out to be multidrug resistant (MDR) all others were sputum smear negative at sixth month of follow-up. Most of the PTB recruited either had history of TB in family or lived in close contact of PTB.

**Table 4:** Conversion of sputum AFB smear^a^ in PTB during ATT.

Categories^b^	At 0 month	At 2 month	At 4 month	At 6 month
High positives (3 + ^c^ and 2 +)	3 + (*n* = 5)	1 + (*n* = 1)	−ve (*n* = 7)	3 + ^d^ (*n* = 1)
		Scanty (*n* = 1)		
		−ve (*n* = 3)		−ve (*n* = 6)
	2 + (*n* = 2)	−ve (*n* = 2)		

Low positives (1 + and scanty)	1 + (*n* = 4)	−ve (*n* = 5)	−ve (*n* = 5)	−ve (*n* = 5)
	Scanty (*n* = 1)			

*^a^The MTB culture results were in concordance with smear microscopy at all the time points of follow-up*.

*^b^Grading of AFB smears as per WHO and IUATLD recommendation*.

*^c^Categories such as high positives and low positives were made based on bacillary load in sputum smear microscopy*.

*^d^The patient was diagnosed to have MDR-TB during sixth month of treatment based on Xpert^®^ MTB-RIF assay (Cepheid, Sunnyvale, CA, USA)*.

*AFB, acid-fast bacilli; PTB, pulmonary tuberculosis patients; ATT, antituberculous treatment, MDR-TB, multidrug resistant TB; MTB, Mycobacterium tuberculosis; WHO, World Health Organization; IUATLD, International Union Against Tuberculosis and Lung Disease*.

### Evaluation of Markers and Cellular Subsets in Peripheral Blood of NHS and Treatment Naïve PTB

We evaluated the expression (in terms of absolute number, percentage, and MFI) (both with and without PPD stimulation) of various Treg and Th17-associated markers (CD4, CD25, CD127, CD39, FoxP3, IL-10, TGF-β and IL-17A) and frequencies/percentages of different cellular subsets (Table 5) in PBMCs isolated from the peripheral blood of PTB (*n* = 15) and NHS (*n* = 17). In NHS, QFT-IT status (i.e., QFT +ve or QFT −ve) had no impact on the expression of these markers.

**Table 5:** Markers and cellular subsets evaluated in the study.

Markers	Cellular subsets	
	Treg subsets	Cellular subsets other than Treg
CD4	CD4^+^CD25^+^FoxP3	CD4^+^CD25^+^
CD25	CD4^+^CD25^+^CD39^+^	CD4^+^CD39^+^
CD39	CD4^+^CD25^+^FoxP3IL-10	CD4^+^CD127^+^
CD127	CD4^+^CD25^+^FoxP3TGF-β	CD4^+^FoxP3
FoxP3	CD4^+^CD25^+^FoxP3CD127^lo^	CD4^+^IL-10
IL-10		CD4^+^TGF-β
TGF-β		CD4^+^IL-17A
IL-17A		CD4^+^CD25^+^CD127^+^
		CD4^+^CD25^+^IL-10
		CD4^+^CD25^+^TGF-β

*Treg, T regulatory cells; FoxP3, forkhead box protein-3; IL-10, interleukin-10; TGF-β, transforming growth factor-beta; IL-17A, interleukin-17A*.

**Figure 2:**
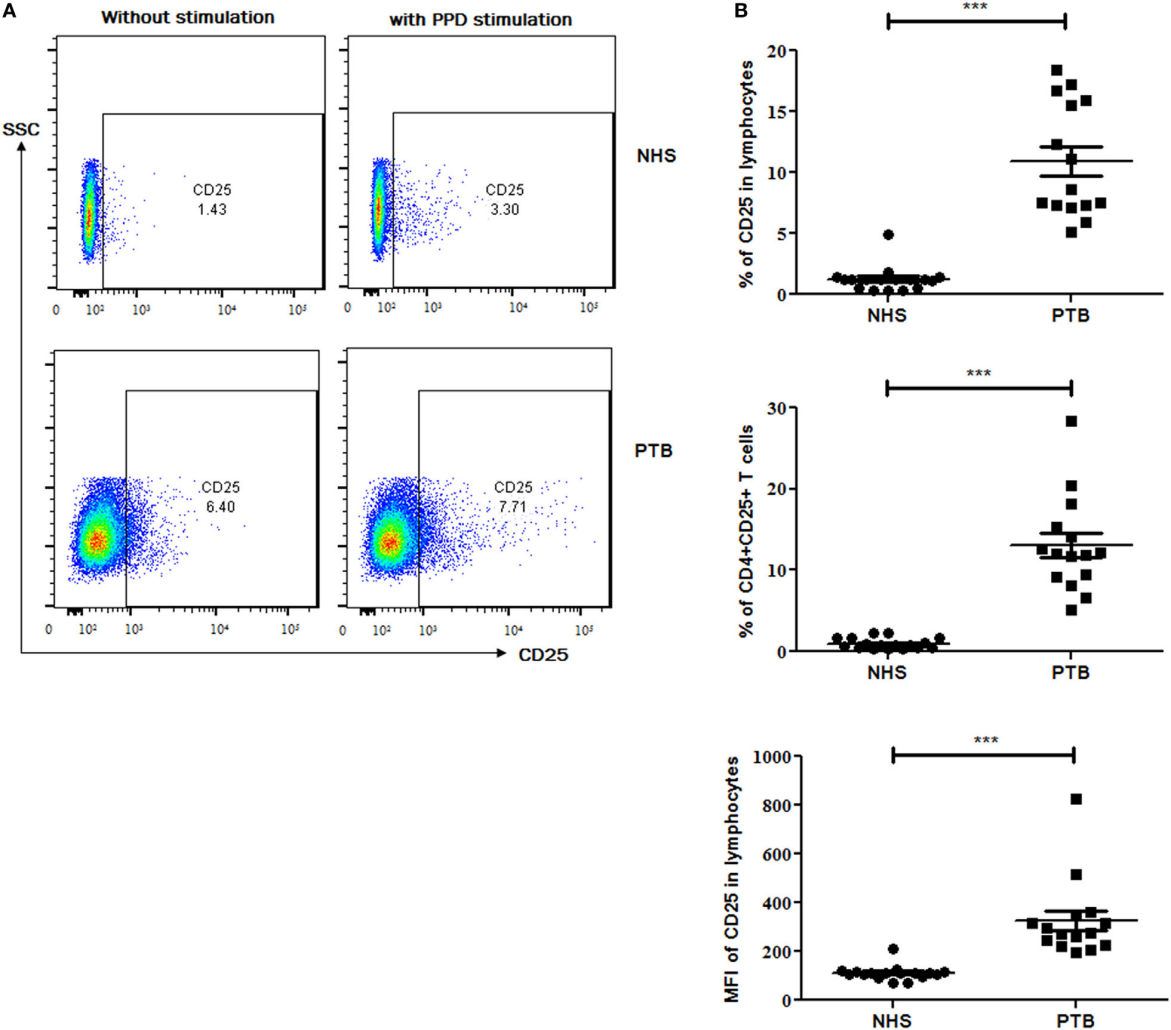
Expression of CD25 marker and cellular subset in peripheral blood of NHS and treatment naïve PTB. **(A)** Flow-cytometry plots (on left) showing the expression of CD25 with and without PPD stimulation and **(B)** scattergrams (on right) between NHS and PTB for CD25 and CD4^+^CD25^+^ T cells. Statistical analysis was performed using Mann–Whitney *U* test. *p ≤ 0.05, **p ≤ 0.01, and ***p ≤ 0.001. NHS, normal healthy subjects; PTB, pulmonary tuberculosis patients; MFI, Mean fluorescence intensity.

**Figure 3:**
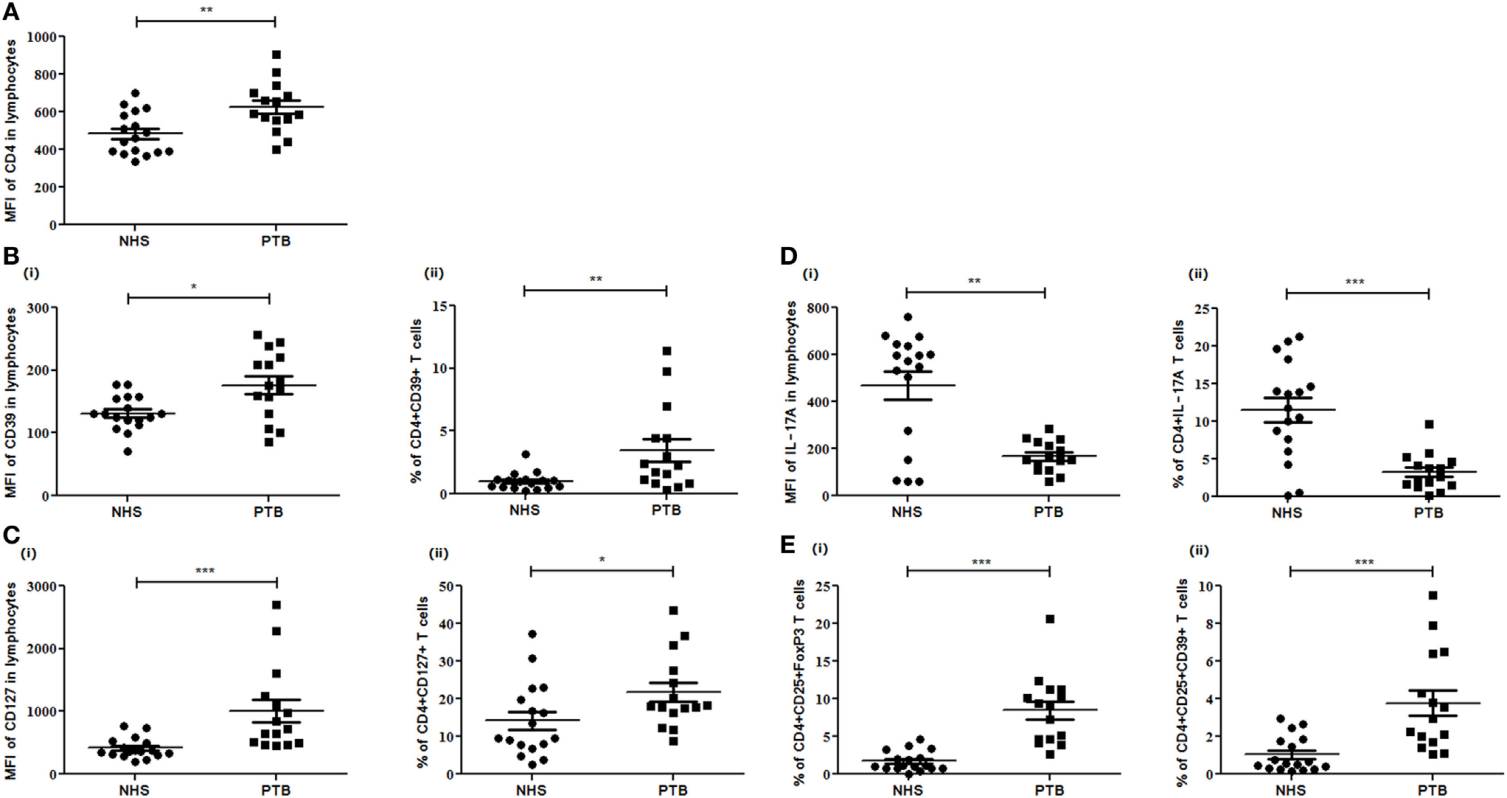
Expression of different markers and subsets in peripheral blood of NHS and PTB. Mean fluorescent intensity (MFI) and percentages of **(A)** CD4, **(B)** CD39 and CD4^+^CD39^+^, **(C)** CD127 and CD4^+^CD127^+^, **(D)** IL-17A and CD4^+^IL-17A, and **(E)** CD4^+^CD25^+^FoxP3 and CD4^+^CD25^+^CD39^+^ between NHS and PTB. Statistical analysis was performed using Mann–Whitney *U* test. *p ≤ 0.05, **p ≤ 0.01, and ***p ≤ 0.001. NHS, normal healthy subjects; PTB, pulmonary tuberculosis patients.

A significantly higher expression of CD25 (*p*<0.0001) (Figure 2), as an individual marker, as well as on CD4^+^ (CD4^+^CD25^+^) (*p*<0.0001) T cells was observed in PTB, when compared with NHS. PPD stimulation had no effect on the expression of CD25 as well as on the frequency of CD4^+^CD25^+^ cells (Figure 2). Markedly higher expression was estimated for CD4 (*p*<0.018), CD39 (*p*<0.028) and CD127 (*p*<0.001) markers (in terms of MFI) as well as CD4^+^CD39^+^ (*p*<0.045) and CD4^+^CD127^+^ (*p*<0.027) cells in PTB when compared with NHS (Figures 3A–C). Interestingly, PPD stimulation further significantly enhanced the expression of the above-mentioned markers and subsets (data not shown). However, the expression of IL-17A (*p*<0.0012) markers and frequencies of CD4^+^IL-17A (*p*<0.0008) T cells were significantly low in PTB than that in NHS (Figure 3Di,ii). The expression of IL-17A was further enhanced significantly on PPD stimulation (data not shown). Rest of the markers and cellular subsets included in the study (as described in Table 5) were also analyzed, but no significant difference was observed between PTB and NHS.

We also enumerated the frequencies of different Treg subsets in PBMCs of PTB and NHS. Peripheral frequencies of CD4^+^CD25^+^FoxP3 (*p*<0.0001), CD4^+^CD25^+^CD39^+^ (*p*<0.0003) Treg subsets were observed to be significantly higher among PTB than that of NHS (Figure 3Ei,ii). No significant difference was observed for induced Treg (iTreg) subsets like CD4^+^CD25^+^FoxP3IL-10 and CD4^+^CD25^+^FoxP3TGF-β when compared between PTB and NHS. Although the frequencies of Treg were enhanced on PPD stimulation, the increase was not significant. Numeral data depicting the absolute count and percentages of various markers and cellular subsets are represented in Table 6.

**Table 6:** Expression of various Treg, Th17 markers, and their cellular subsets (those showing significance) in terms of their absolute number and percentages during recruitment of study subjects.

Markers/subsets		PTB (*n* = 15)	NHS (*n* = 17)
CD25	Ab. no.	221.2 ± 25.64	26.82 ± 5.27
	%	13.10 ± 1.51	1.55 ± 0.30

IL-17A	Ab. no.	85.00 ± 17.39	245.30 ± 30.40
	%	4.92 ± 1.00	14.18 ± 1.75

CD4^+^CD25^+^	Ab. no.	225.5 ± 26.22	16.71 ± 2.76
	%	13.03 ± 1.52	0.98 ± 0.16

CD4^+^CD39^+^	Ab. no.	59.67 ± 15.25	17.29 ± 2.97
	%	3.45 ± 0.88	0.99 ± 0.17

CD4^+^CD127^+^	Ab. no.	375.7 ± 44.05	246.7 ± 41.13
	%	21.72 ± 2.55	14.26 ± 2.38

CD4^+^IL-17A	Ab. no.	56.66 ± 10.97	199.4 ± 27.22
	%	3.27 ± 0.63	11.52 ± 1.58

CD4^+^CD25^+^Foxp3	Ab. no.	69.80 ± 13.59	15.29 ± 2.63
	%	9.79 ± 1.37	2.48 ± 0.45

CD4^+^CD25^+^CD39^+^	Ab. no.	20.67 ± 4.63	5.47 ± 1.26
	%	3.77 ± 0.68	1.04 ± 0.23

*PTB, pulmonary tuberculosis patients; NHS, normal healthy subjects; %, percentages; Ab. no., absolute number*.

*The values represented in the above table are mean ± SEM*.

**Figure 4:**
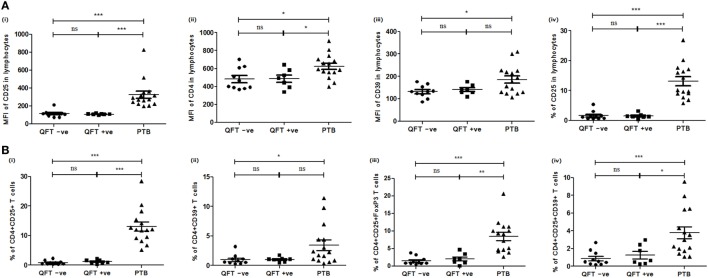
Expression of various markers and cellular subsets in PBMCs of QFT –ve (NHS), QFT +ve (NHS), and PTB. **(A)** Mean fluorescent intensity (MFI) of (i) CD25, (ii) CD4, (iii) CD39 markers in lymphocytes, and (iv) percentage of CD25 T cells in lymphocytes. **(B)** Frequencies of (i) CD4^+^CD25^+^, (ii) CD4^+^CD39^+^ T cells, (iii) CD4^+^CD25^+^FoxP3, and (iv) CD4^+^CD25^+^CD39^+^ Treg. Statistical analysis was performed using Mann–Whitney *U* test *p ≤ 0.05, **p ≤ 0.01, and ***p ≤ 0.001. PBMCs, peripheral blood mononuclear cells; QFT, quantiferon; PTB, pulmonary TB patients; ns, not significant.

Further, based on QFT status, NHSs were categorized into two sub groups: QFT positive (QFT +ve; LTBI, *n* = 7) and QFT negative (QFT –ve; *n* = 10), and the expression of various Treg and Th17 markers and cellular subsets were compared with that of PTB. Similar to the above-mentioned results comparing PTB with NHS, a significantly higher expression of CD4 (QFT +ve, *p*<0.024; QFT –ve, *p*<0.018), CD25 (QFT +ve, *p*<0.0002; QFT –ve, *p*<0.0001), and CD39 (QFT +ve, *p*<0.138; QFT –ve, *p*<0.043) markers and CD4^+^CD25^+^ (QFT +ve, *p*<0.0002; QFT –ve, *p*<0.0001), CD4^+^CD39^+^ (QFT +ve, *p*<0.077; QFT –ve, *p*<0.014), CD4^+^CD25^+^FoxP3 (QFT +ve, *p*<0.001; QFT –ve, *p*<0.0001) and CD4^+^CD25^+^CD39^+^ (QFT +ve, *p*<0.016; QFT –ve, *p*<0.0008) (Figure 4) Treg subsets were observed in PTB when compared with QFT +ve or QFT –ve individuals. However, other markers and cellular subsets also showed a similar trend but were not statistically significant when QFT positive or negative NHS groups were compared with PTB (data not shown).

When the marker expression studies were done in QFT positive and negative subjects (only NHS group), the following results were observed: the expression of various Treg and Th17 markers and percentages of their cellular subsets were compared among QFT +ve individuals, as well as between QFT +ve and QFT −ve individuals, but no significant difference was observed for any of the markers and cellular subsets studied.

### Frequencies of Markers and Cellular Subsets during ATT

**Figure 5:**
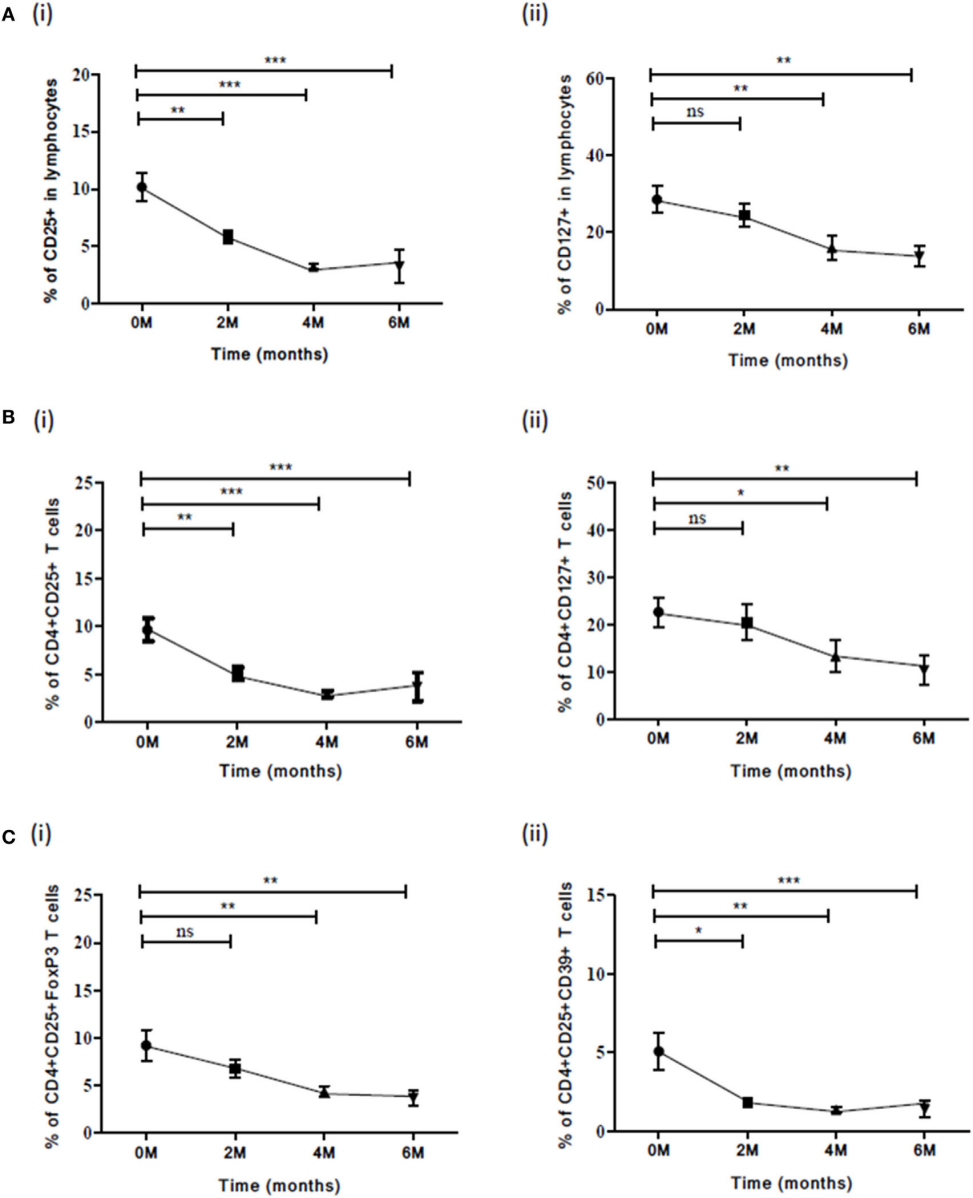
Frequency of various Treg subsets and associated markers at different time points (0, 2, 4, and 6) of TB treatment. **(A)** Expression of (i) CD25 and (ii) CD127 in lymphocytes along with ATT. **(B)** Frequencies of (i) CD4^+^CD25^+^ and (ii) CD4^+^CD127^+^ T cells **(C)** Frequencies of Treg subsets: (i) CD4^+^CD25^+^FoxP3 and (ii) CD4^+^CD25^+^CD39^+^ during ATT. Statistical analysis was performed using Kruskal–Wallis non-parametric test and comparison between the different time points (0–2, 0–4, and 0–6) was made using Mann–Whitney *U* test. *p ≤ 0.05, **p ≤ 0.01, and ***p ≤ 0.001. M, months; ns, not significant; ATT, antituberculous treatment.

We evaluated the expression of various Treg and Th17 markers and frequencies of cellular subsets (Table 5) in PBMCs of PTB (at different time points of ATT) for determining the role of these markers in TB treatment efficacy. In this context, 12 PTB were followed up till ATT completion. The frequencies of CD25 (*p*<0.0001) and CD127 (*p*<0.025) marker decreased over the course of treatment (Figure 5Ai,ii). A decrease was also observed in frequencies of cellular subsets like CD4^+^CD25^+^ (*p*<0.0001), CD4^+^CD127^+^ (*p*<0.006) T cells (Figure 5Bi,ii) and Treg subsets like CD4^+^CD25^+^FoxP3 (*p*<0.038) and CD4^+^CD25^+^CD39^+^ (*p*<0.005) during ATT (Figure 5Ci,ii). However, no decrease in frequencies of iTreg—CD4^+^CD25^+^FoxP3IL-10 and CD4^+^CD25^+^FoxP3TGF-β was observed. PPD stimulation had no effect on expression of these markers and did not alter the overall significance (meaning that at least median of one time point is different from the median of at least any other time point). No significant variation was observed for other markers and subsets during ATT.

In the case of one PTB who turned out to be MDR during the sixth month, a drastic increase was observed only in CD25 expression and CD4^+^CD25^+^ subset frequency when compared with other PTB (Table 7).

**Table 7:** Comparison between the expression of CD25 marker and its CD4^+^ subset in a PTB with that of a PTB who turned into MDR.

Marker/cellular subset	Patient ID	Percentages of different time points of follow-up			
		0M	2M	4M	6M
CD25	Tn-01^a^	7.30	4.18	2.24	18.40
	Tn-05	5.93	5.84	2.54	2

CD4^+^CD25^+^	Tn-01^a^	17.5	5.49	1.95	19.70
	Tn-05	9.23	7.80	2.86	1.88

*^a^PTB who turned into MDR at sixth month of follow-up*.

*PTB, pulmonary tuberculosis patients; M, month; MDR, multidrug resistant*.

### Role of the Markers and Cellular Subsets in Early Treatment Monitoring

**Figure 6:**
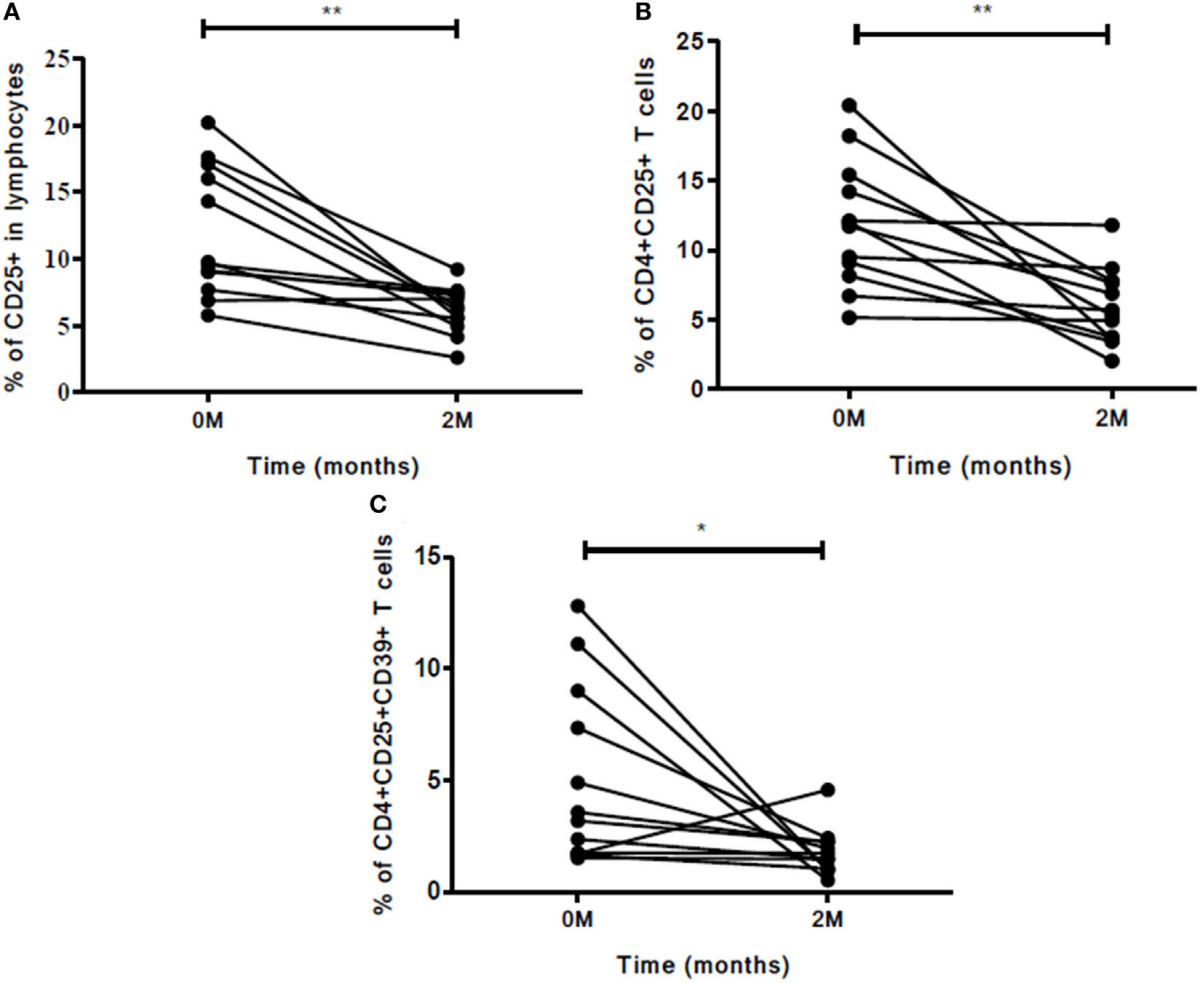
Frequencies of Treg and associated markers for early (at second month) treatment monitoring. **(A)** Percentage of CD25 marker, **(B)** percentage of CD4^+^CD25^+^ T cells, and **(C)** frequency of CD4^+^CD25^+^CD39^+^ Treg. The change in expression of markers and cellular subsets was studied between zero and second months of ATT. Statistical analysis was made using Mann–Whitney *U* test. *p ≤ 0.05, **p ≤ 0.01, and ***p ≤ 0.001. Other markers and cellular subsets were also studied but were not found to be significant. M, months; ATT, antituberculous treatment; Treg, T regulatory cells.

The role of these markers as well as cellular subsets was studied at zero and second months of ATT for early treatment monitoring. The frequencies of CD25 (*p*<0.01) marker decreased significantly at the end of IP (Figure 6A). The cellular subsets like CD4^+^CD25^+^ (*p*<0.001) and CD4^+^CD25^+^CD39^+^ (*p*<0.05) (Figures 6B,C) also decreased significantly at second month of ATT. However, for rest of the markers no significant difference in expression from zero to second month was noticed. Also, stimulation with PPD did not change the expression of markers and cellular subsets at early time point of treatment.

### Role of Markers and Subsets in Determining Treatment Efficacy during Continuation Phase (CP) of Treatment

**Figure 7:**
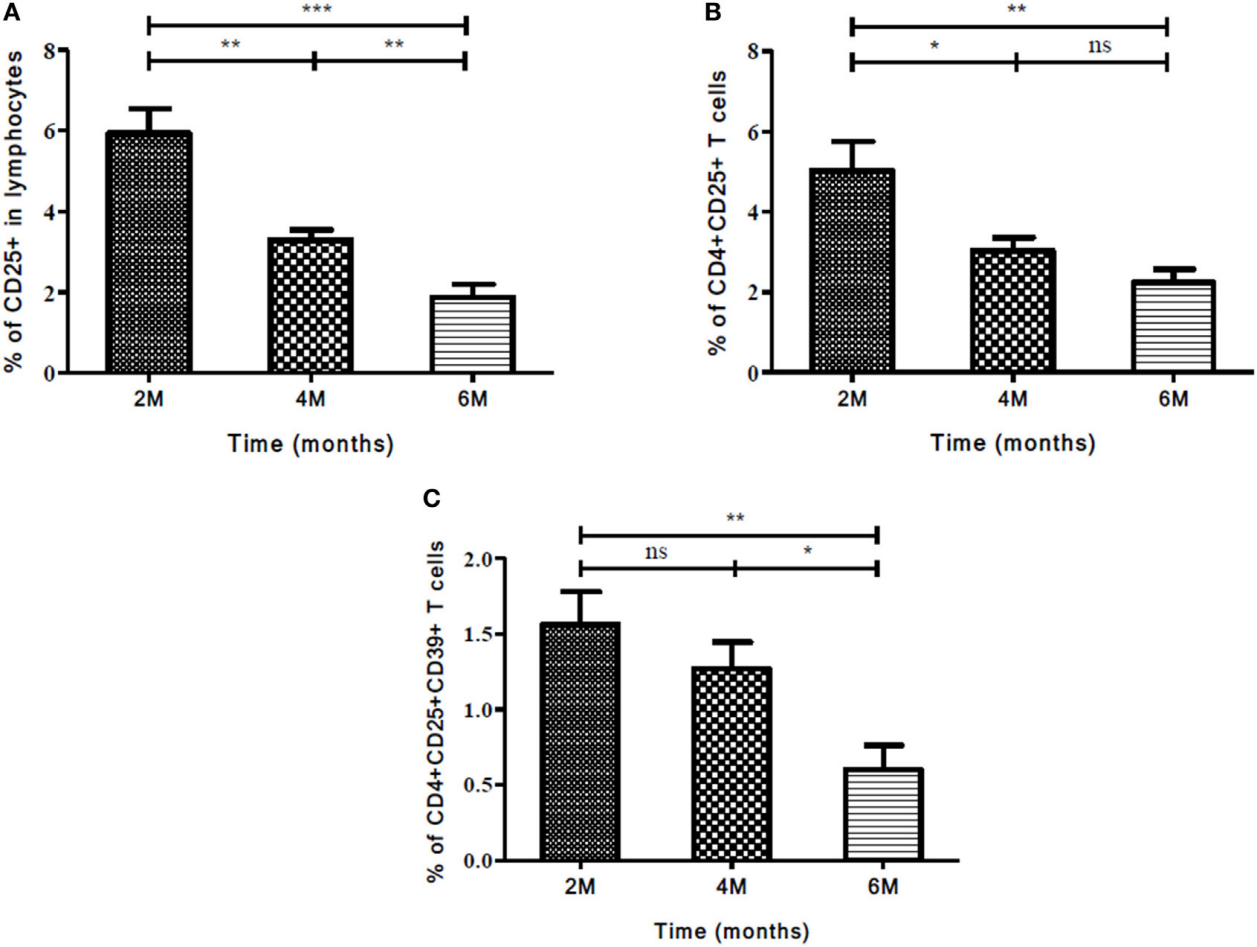
Frequency of Treg and associated markers during continuation phase (CP) of TB treatment. **(A)** Expression of CD25 as an individual marker, **(B)** frequency of CD4^+^CD25^+^, and **(C)** CD4^+^CD25^+^CD39^+^ T cells at different time points (2, 4, and 6 months) of treatment. Mann–Whitney *U* test was used to measure statistical significance between various time points like 2 and 4 months, 2 and 6 months, and 4 and 6 months. *p ≤ 0.05, **p ≤ 0.01, and ***p ≤ 0.001. M, months; ns, not significant; Treg, T regulatory cells.

The expression of Treg and Th17 markers and cellular subsets were evaluated in CP of ATT. The expression of these markers was compared between second and fourth months; second and sixth months; fourth and sixth month. A significant decrease was observed in the expression of CD25 marker, CD4^+^CD25^+^, and CD4^+^CD25^+^CD39^+^ T cells at all treatment time point comparisons mentioned above (Figures 7A–C). Stimulation with PPD did not change the expression of markers and cellular subsets in CP of ATT.

### Association of Markers and Cellular Subsets with Sputum Conversion at the End of Intensive Phase (IP)

**Figure 8:**
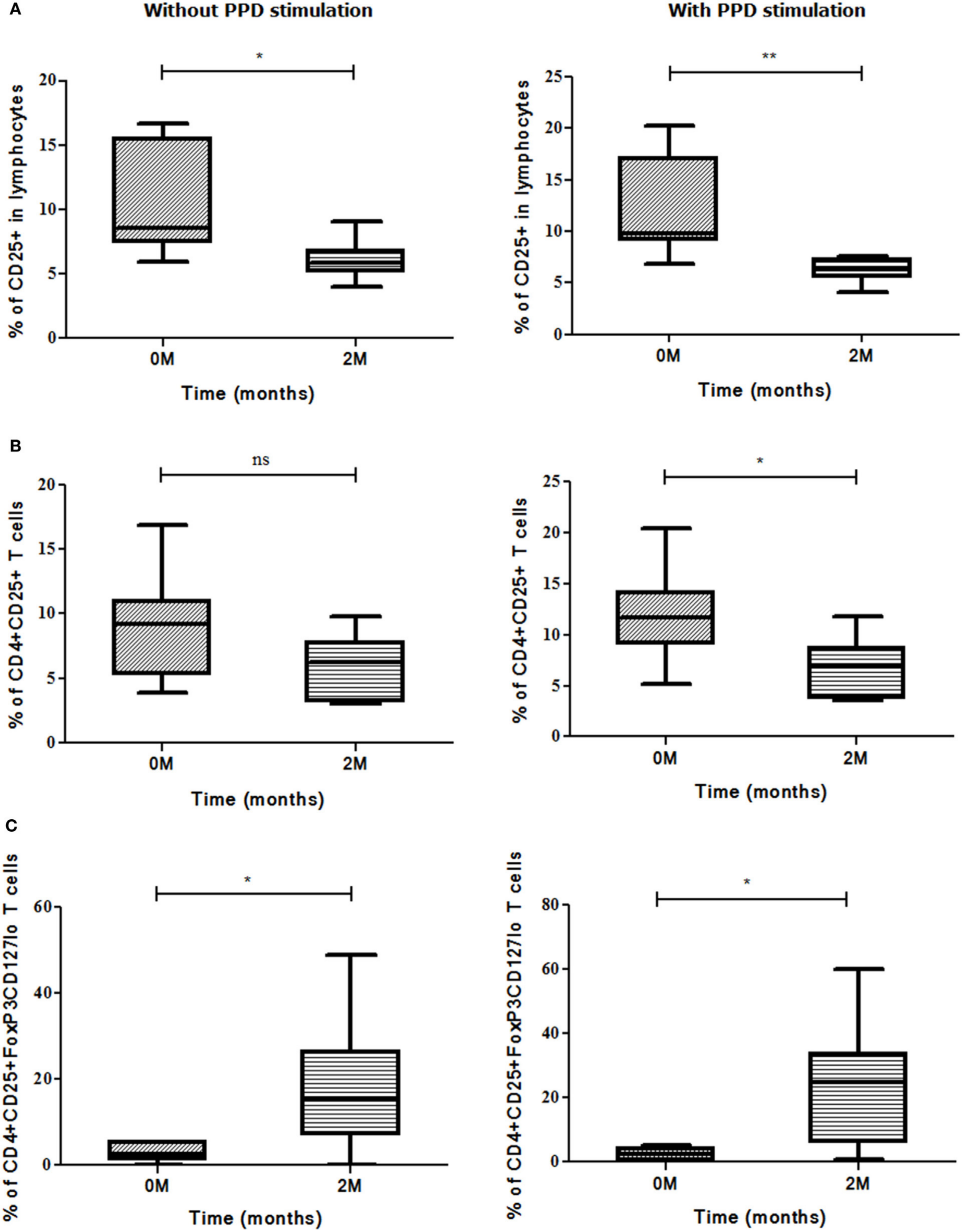
Sputum conversion in high positives at the end of intensive phase (IP). Frequencies of **(A)** CD25, **(B)** CD4^+^CD25^+^ T cells, and **(C)** CD4^+^CD25^+^FoxP3CD127^lo^ Treg (with and without stimulation) along with sputum conversion in high positives (3 + and 2 +) at zero and second months. Sputum conversion and frequencies of markers were statistically correlated by employing Mann–Whitney *U* test. *p ≤ 0.05, **p ≤ 0.01, and ***p ≤ 0.001. M, months; Treg, T regulatory cells; ns, not significant; PPD, purified protein derivative.

It was observed that higher the bacillary load, higher the expression of evaluated markers and vice versa but this was not statistically significant (data not shown). The bacillary load reduction assessed by second month (IP) sputum smear conversion was investigated along with different marker levels for deducing an asssociation, if any. In sputum high positives (sputum smear grades: 3 + or 2 + during recruitment), the frequencies of CD25 (*p*<0.017) marker and CD4^+^CD25^+^ (*p*<0.02) T cells decreased significantly along with the conversion of sputum bacillary load at the end of IP (Figures 8A,B). On the other hand, an inverse association was found between change in bacillary load from zero to second months and CD4^+^CD25^+^FoxP3CD127^lo^ (*p*<0.011) Treg frequency (Figure 8C). PPD stimulation enhanced the expression of above-said markers and subsets significantly in high smear positive sputum converters at second month (Figures 8A–C).

**Figure 9:**
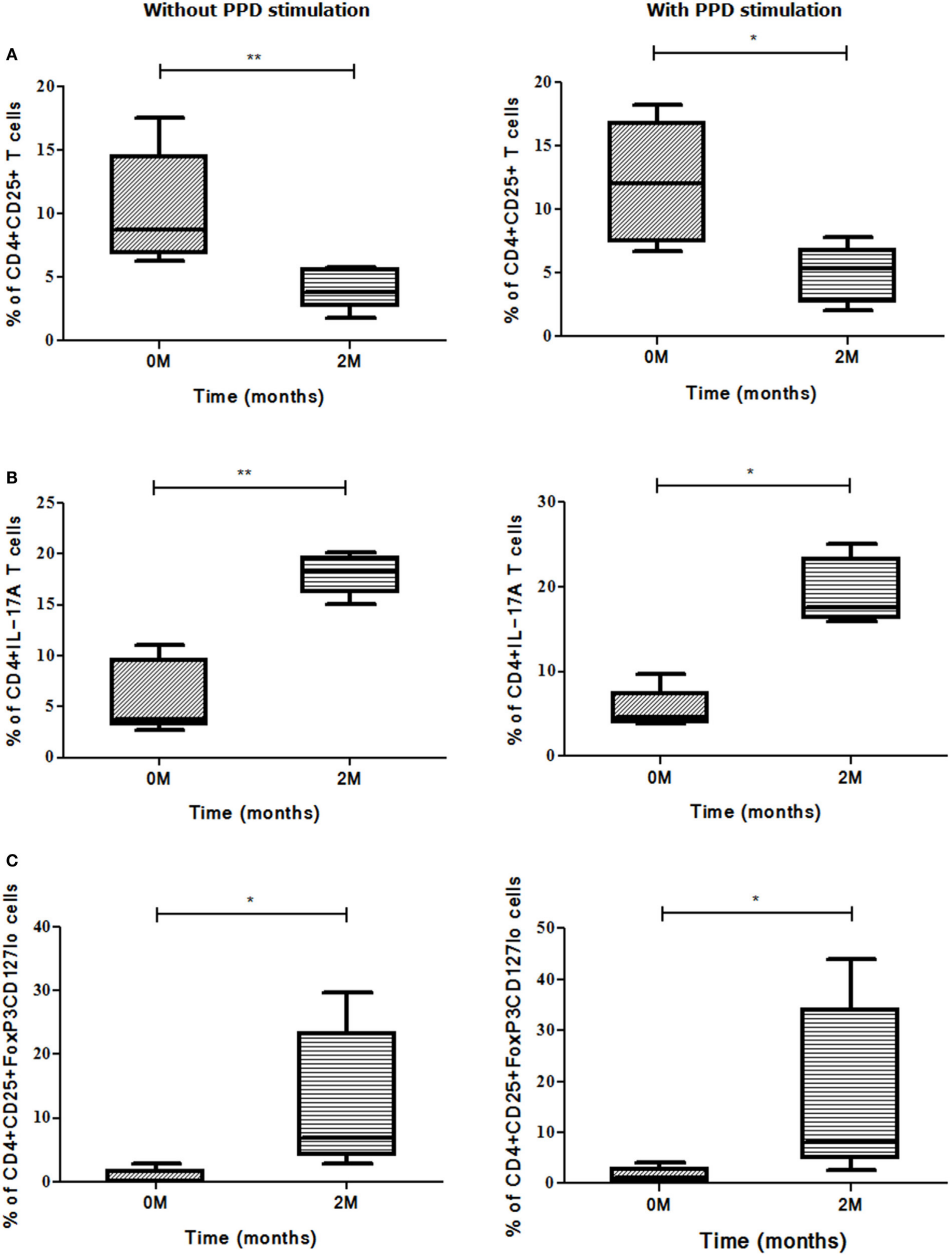
Sputum conversion in low positives (1 + and scanty) at the end of intensive phase (IP). Association was deduced between frequencies of **(A)** CD4^+^CD25^+^, **(B)** CD4^+^IL-17A, and **(C)** CD4^+^CD25^+^FoxP3CD127^lo^ Treg and sputum conversion among low positives from zero and second months using Mann–Whitney *U* test. *p ≤ 0.05, **p ≤ 0.01, and ***p ≤ 0.001. M, months; ns, not significant; Treg, T regulatory cells; PPD, purified protein derivative.

A positive association was found between second month smear conversion of sputum low positives (sputum smear grades: 1 + or scanty during recruitment) and frequencies of CD4^+^CD25^+^ (*p*<0.008) T cells, whereas the frequencies of CD4^+^IL-17A (*p*<0.008) T cells and CD4^+^CD25^+^FoxP3CD127^lo^ (*p*<0.011) Treg showed an inverse association (Figures 9A–C). PPD stimulation enhanced the expression of the above markers and subsets but was not significant.

### Radiological Parameters, Bacillary Load and Marker Studies

**Figure 10:**
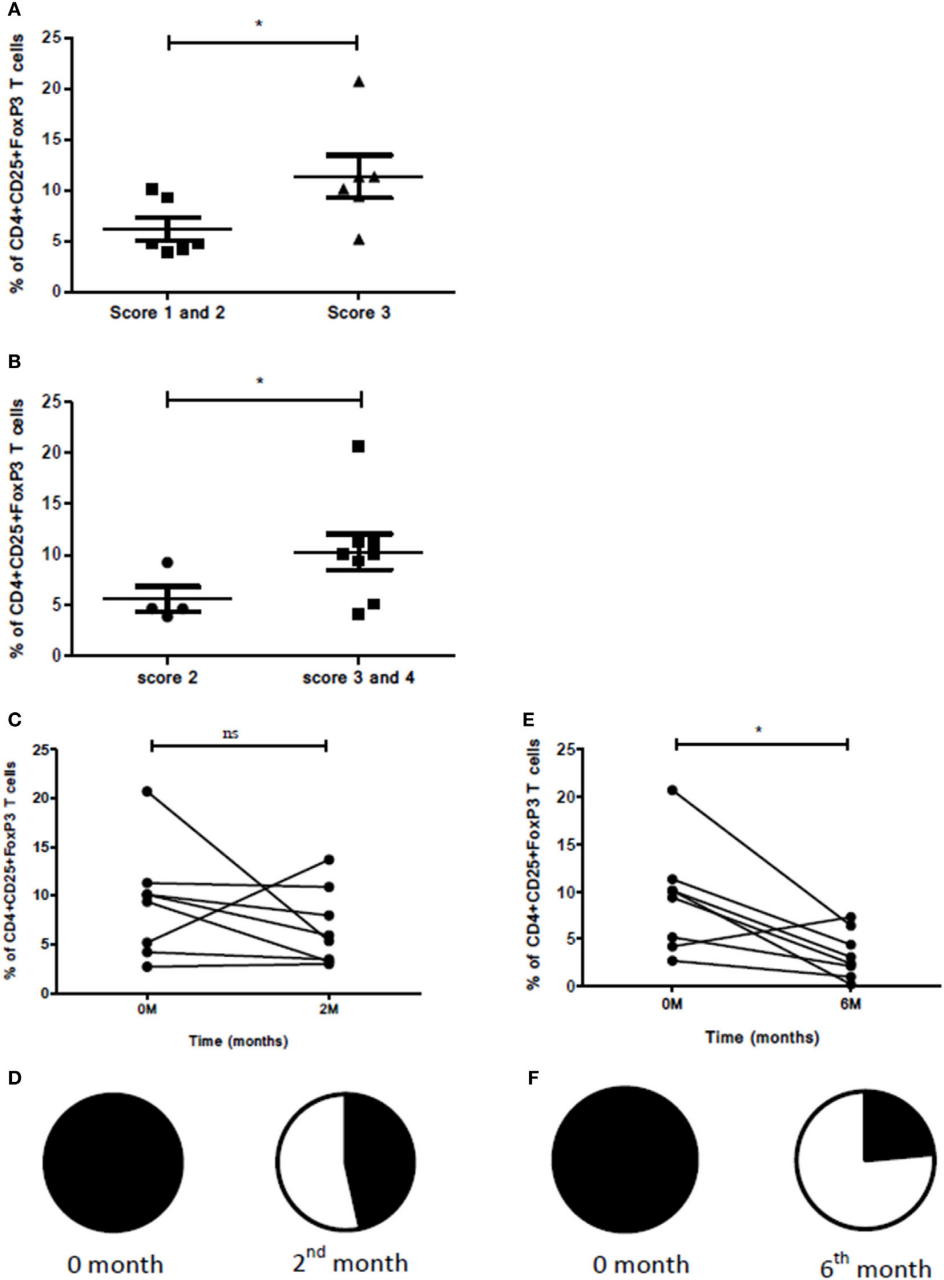
Association between radiological scores, sputum smear grades, and Treg levels. **(A)** Bacillary load, number of lesions, and percentage of T cells expressing CD4^+^CD25^+^FoxP3. Here, score 1––patients having sputum smear grade scanty/1 + with single lesion (*n* = 1); score 2––patients having sputum smear grade scanty/1 + with multiple chest X-ray lesions (*n* = 5); score 3–patients having sputum smear grade 2 + /3 + with multiple chest X-ray lesions (*n* = 6). **(B)** Association between type of lesions and percentage of T cells expressing CD4^+^CD25^+^FoxP3. Here, score 2––patients having opacity (*n* = 4); score 3––patients with opacity and single cavity (*n* = 5); and score 4––patients with opacity and multiple cavities (*n* = 3). **(C)** Reduction in percentage of cells expressing CD4^+^CD25^+^FoxP3 before ATT and 2 months after ATT [in eight patients having opacity with single and multiple cavities (score 3 and 4)]. **(D)** Pie chart denoting reduction in chest X-ray lesions 2 months after ATT for eight patients shown in **(C)**. Closed segment denotes no improvement and open segment denotes improvement in chest X-ray lesions. **(E)** Reduction in percentage of cells expressing CD4^+^CD25^+^FoxP3 before ATT and 6 months after ATT for eight patients shown in **(C)**. **(F)** Pie chart denoting reduction in chest X-ray lesions 6 months after ATT for eight patients shown in **(C)**. M, months; ATT, antituberculous treatment.

Of all the markers and cellular subsets studied, the percentage of CD4^+^CD25^+^FoxP3 Treg subsets showed a significant increase in those who manifested high bacillary load and multiple lesions by radiology when compared with those who had low bacillary load and single/multiple lesions (Figure 10A). The same subset also showed a significant increase in patients with opacities and single or multiple cavities (scores 3 and 4; *n* = 5, 3) compared with those who had opacities alone (score 2; *n* = 4) in chest X-rays (Figure 10B). A decrease of CD4^+^CD25^+^FoxP3 Treg subset along with reduction in chest X-ray lesion was observed in the second month but was not statistically significant (Figures 10C,D). When this Treg subset was studied in follow-up (second and sixth month compared with zero month), it manifested a significant decrease in the sixth month (*p*<0.018) with a concomitant reduction in chest X-ray lesions (Figures 10E,F). The radiological parameters were studied for all the other markers and cellular subsets wherein a change was observed for the scores compared but was not statistically significant.

## Discussion

Efficient treatment is crucial for TB disease control. Although the current regimens are for extended periods of time (from months to years), patient compliance and prompt monitoring will help in efficient management. Monitoring ATT is necessary as late detection of resistance (due to non-compliance) may lead to further complications (like poor prognosis) and extended therapy for the patient which is more burdensome for the patient’s health as well as for the country’s economy. Currently, monitoring ATT involves sputum microscopy, demerits of which have been described earlier (2, 3). An efficient monitoring tool is the need of the hour and earlier studies have focused on serum markers like IL-2 (12), neopterin (12), IL-1ra (13), C-reactive protein (13), sTNF-R1 (14), granzyme B (14), and cell-associated markers like CD4^+^CD38^+^ (15), CD4^+^HLA-DR (15, 16), CD4^+^Ki-67 (15), CD11b, CD33 (studied as part of granulocytic and monocytic MDSCs) (16), CD4^+^CD25^+^ (4, 17, 18), CD4^+^CD25^+^FoxP3 (5, 6, 19, 20), and CD4^+^IL-17A (21–23). Serum markers studied so far are not promising as they have low predictive values and are not above the baseline. Although cells are dynamic structures, producing markers (both on the surface and internally) which have a turnaround time, their expression follows a definite pattern during treatment which can be exploited for early treatment monitoring and studying treatment efficacy during ATT (4, 5, 14–18, 24).

The role of Treg in TB is contradictory, as it is unclear whether the peculiar expansion of Treg is a cause or consequence of disease. This expansion is probably in retort to the enhanced pro-inflammatory immune response (by Th1 and Th17 subsets) for bringing down the immune-mediated damage. There are reports pointing out the involvement of Treg and Th17 in TB disease progression (20–23), severity of TB (25–28), and even reports showing a regular gradation in frequencies of Treg in response to ATT (4, 5, 17–19, 24). But none of these studies have comprehensively evaluated the dynamics of various Treg and Th17 cellular subsets and their associated markers along with ATT (at various time points of TB treatment i.e., before, during, and after treatment). In this study, we have evaluated (i) dynamics of various Treg and Th17 subsets and associated markers at various time points of ATT (0, 2, 4, and 6) and their use in monitoring ATT; and (ii) their association with sputum conversion.

The initial part of this study has compared the expression of Treg and Th17 markers and cellular subsets between NHS and treatment naïve PTB. A higher expression of CD25 as an individual marker as well as on CD4^+^ (CD4^+^CD25^+^) T cells was observed in PTB than those from NHS, which is in agreement with the earlier studies (17, 18). It is known that CD25 (IL-2Rα) is indispensable for the maintenance of FoxP3 expression as well as for thymic and extrathymic differentiation of Treg subsets (29, 30). Thus, Treg cells express abundant amount of IL-2Rα. Despite their higher dependence on IL-2, Treg cells are unable to produce IL-2 and rely on IL-2 produced by activated T cells. The IL-2 consumption by Treg is suggested to play an essential role in Treg suppressor function by causing death of activated CD4^+^ T cells due to IL-2 deprivation (31, 32). This could be the reason for enhanced CD25^+^ (IL-2Rα) marker expression individually and also on CD4^+^ cells (CD25^+^ has weak affinity for IL-2).

Furthermore, the frequency of CD4^+^CD25^+^FoxP3 and CD4^+^CD25^+^CD39^+^Treg was higher in peripheral blood of PTB when compared with NHS, which is found to be in concordance with previous studies (4, 5, 33). FoxP3 is considered a key player for Treg development and function, whereas CD39 is an ectoenzyme that degrades adenosine triphosphate (ATP) to adenosine that inhibits effector T-cell activation, proliferation, and expansion. It has been shown to be associated specifically with Treg function. Therefore, a higher frequency of these Treg subsets is commonly observed in response to many infections including MTB infection.

On the contrary, PTB had a lower expression of Th17 cells (IL-17A and CD4^+^IL-17A T cells) which was reciprocal to CD4^+^CD25^+^FoxP3 expression when compared with NHS. Our results correspond with the results by Shu et al. (23), who found a reciprocal relation in frequencies of Th17 and CD4^+^CD25^+^FoxP3 Treg in TB patients when compared with controls. This imbalance in frequencies of Th17 and Treg may be because they have a mutually inhibitory relationship in terms of differentiation, development and function (34).

Our study results also suggest that cellular markers like CD25, CD4^+^CD25^+^, CD4^+^CD25^+^FoxP3 and CD4^+^CD25^+^CD39^+^ are able to discriminate between latent and active TB after using PPD as antigenic stimulus. This is of utmost importance as markers for differential diagnosis of latent infection and active TB disease are still sought for. In line with our findings, Chiappini et al. (35) have found that a combination of IL-2 and IFN-γ-based ELISPOT can distinguish LTBI and active TB disease in pediatric population.

Our study result adds on to the existing knowledge of Treg expansion during TB. The elevated Treg are associated with immune suppression and their frequencies are supposed to decrease subsequently along with treatment as shown by other studies (discussed below). Thus, these marker levels will be helpful in monitoring TB treatment response.

In this context, our data demonstrate a marked decrease in the expression of CD25, CD127 markers, CD4^+^CD25^+^, CD4^+^CD127^+^ T cells, and Treg subsets like CD4^+^CD25^+^FoxP3 and CD4^+^CD25^+^CD39^+^along with treatment. Such a decrease in expression of markers and cellular subsets was not confined only to the end of IP but was consistent throughout the treatment even after IP and during CP. Our data correspond with other existing reports on decrease of Treg subsets but none of those studies have examined both Treg and Th17 subsets comprehensively till the end of ATT. Ribeiro-Rodrigues et al. (17) observed a significant drop in frequencies of CD4^+^CD25^+^ T cells at week 24 of ATT. Similarly, Singh et al. (5) found a decrease in frequencies of CD4^+^CD25^+^FoxP3 Treg after successful ATT. He et al. (18) also observed a decrease of CD4^+^CD25^+^FoxP3 Treg just after one month of treatment, whereas no difference was observed for CD4^+^CD25^+^ T cells. Additionally, Jackson-Sillah et al. (19) have also found a progressive decrease in frequencies of CD4^+^CD25^+^FoxP3 Treg at the end of second month of ATT.

In our study, a reduction in frequencies of Treg and Th17 markers and cellular subsets mirroring sputum conversion has been observed at the end of second month of ATT. A significant decrease in the frequency of CD4^+^CD25^+^ T cells, both in sputum high and low positives, has been observed upon sputum conversion from zero to second month of follow-up of PTB. Thus, ATT induced a decrease in Treg frequencies suggestive of their MTB-driven expansion at initial time point which later decrease with successful clearance of bacilli. On the other hand, the Th17 subset showed a marked increase in frequency upon sputum conversion which supports the theory of reciprocal expression of Th17 and Treg (36).

In one of the PTB who turned out to be MDR at sixth month of follow-up, we observed an abrupt increase in frequencies of CD25 marker and CD4^+^CD25^+^ T cells which is in line with the previous reports of expansion of Treg in MDR TB (27, 37). Furthermore, Geffner et al. (37) found increased frequencies of CD4^+^CD25^+^ and CD4^+^CD25^high^FoxP3 Treg in MDR-TB compared with susceptible-TB and healthy controls. A similar increase in frequency of CD4^+^CD25^+^FoxP3 Treg has been observed in MDR-TB by another study (27).

Although many studies have been carried out relating to Treg, Th17 with TB disease, this study is unique in that a real-time association between Treg and Th17 markers and the course of disease during treatment has been done. In this study, a detailed analysis of all the markers and their association with sputum microscopy at all the time points of treatment (0, 2, 4, and 6 months) has been done to know their usefulness in treatment monitoring. The overall aim of this study was to investigate multiple markers and cell subsets related to Treg and Th17 subsets and find out markers whether single or as a cellular subset which can be useful in monitoring TB treatment. Thus, the strength of this study is the evaluation of multiple markers and cellular subsets during ATT but the validation needs to be performed in a large cohort of PTB using multiple antigens. Thus, from this study, it was observed that markers like CD25, CD4^+^CD25^+^, and CD4^+^CD25^+^CD39^+^ T cells are distinct in their elevated expression in PTB before treatment when compared with treatment completion. Moreover, they show a consistent decrease in IP as well as in CP showing their potential for monitoring TB treatment efficacy. When the efficiency of these markers in comparison to sputum microscopy was studied, only CD25 and CD4^+^CD25^+^ T cells were able to show a consistent performance in high-positive as well as low-positive sputum converters. When treatment success at sixth month was compared with the level of markers and cell subsets, only a decrease in CD4^+^CD25^+^FoxP3 T-cell subset showed a statistically significant association. The decrease in the level of CD4^+^CD25^+^FoxP3 cells during treatment also showed good association with resolution of extensive lung lesions. Thus, this Treg subset appears to be promising for predicting favorable treatment response in PTB with extensive lung lesions. All these characteristics point out that these markers deserve attention for use in TB treatment monitoring.

## Ethics Statement

The study was carried out after obtaining approval from Institutional Human Ethics Committee (IHEC). All study participants provided written informed consent.

## Author Contributions

SA performed the experiments, analyzed the data, and prepared the manuscript. MK prepared the manuscript and analyzed the data. OP and MK designed the study and participated in data interpretation. AP made critical review of the manuscript and helped in modifying the manuscript. AP also contributed toward analyzing the clinical data and in its interpretation. AB helped in manuscript preparation and data analysis. SK helped in interpreting the clinical data. DC helped in microbiological studies.

## Conflict of Interest Statement

The authors declare that the research was conducted in the absence of any commercial or financial relationships that could be construed as a potential conflict of interest.

## Supplementary Material

Figure S1 Recruitment of study groups and the experimental methodology. Sputum and blood samples were collected from both PTB. Sputum was subjected to smear microscopy and MTB culture, whereas blood was subjected to T-cell immune assay. TB, tuberculosis; PTB, pulmonary TB patients; ATT, antituberculous treatment; MTB, *Mycobacterium tuberculosis*; AFB, acid-fast bacilli.
