# Supplementary figures and images for: Efficacy of T Regulatory Cells, Th17 Cells and the Associated Markers in Monitoring Tuberculosis Treatment Response
Source: Front Immunol. 2018 Feb 5;9:157. doi: 10.3389/fimmu.2018.00157 (PMC5810270; doi:10.3389/fimmu.2018.00157)

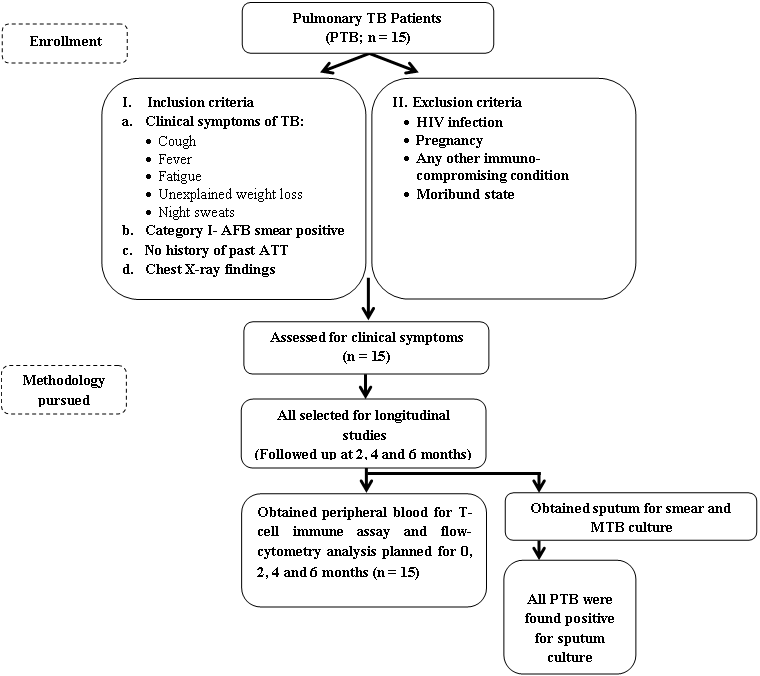

Supplement: Figure S1 — Recruitment of study groups and the experimental methodology. Sputum and blood samples were collected from both PTB. Sputum was subjected to smear microscopy and MTB culture, whereas blood was subjected to T-cell immune assay. TB, tuberculosis; PTB, pulmonary TB patients; ATT, antituberculous treatment; MTB, Mycobacterium tuberculosis; AFB, acid-fast bacilli. [file Image_1.tif]
